# Battle with COVID-19 in Iran: What lessons can be learned from the implementation of response strategies so far?

**DOI:** 10.1017/ice.2020.231

**Published:** 2020-05-13

**Authors:** Mehrdad Amir-Behghadami, Ali Janati, Masoumeh Gholizadeh

**Affiliations:** 1 Tabriz Health Services Management Research Center, Health Management and Safety Promotion Research Institute, Tabriz University of Medical Sciences, Tabriz, Iran; 2 Iranian Center of Excellence in Health Management (IceHM), School of Management and Medical Informatics, Tabriz University of Medical Sciences, Tabriz, Iran; 3 Student Research Committee (SRC), Tabriz University of Medical Sciences, Tabriz, Iran

*To the Editor*—The novel coronavirus disease (COVID-19), with human-to-human transmission and severe human infection, has been escalating rapidly since late December 2019.^1^ Disease symptoms can range from mild flu-like cases to severe cases with life-threatening pneumonia.^2,3^ The global condition is evolving dynamically, and on January 30, 2020, the World Health Organization (WHO) announced that COVID-19 is a “public-health emergency of international concern.” During the coronavirus pandemic, the authorities of the Iranian Ministry of Health and Medical Education (MOHME) reported the first cases of coronavirus on February 19, 2020 in Qom.^4^ As of March 6, 2020, according to MOHME, 27,017 cases of COVID-19 have been identified in the country, 2,077 of whom have died and 9,625 of whom have recovered so far. Following the widespread outbreak of SARS-CoV-2 in China, the MOHME launched a campaign in early February 2020 including monitoring and examining all incoming travelers from China and quarantine of Iranian students residing in China.

Currently, no licensed vaccine for specific antiviral prevention and treatment is available for COVID-19.^5^ Therefore, the most effective measures are to eliminate the source of infection, to cut off the transmission route and to protect the susceptible.^6^ Prevention and control became the most urgent task in Iran during the early days of the sudden outbreak of the SARS-CoV-2 virus.^7,8^ In this regard, the government has invested large amounts of human capital and material resources. Regarding the origin of the infection, people who are in close contact with patients may become new patients or new sources of infection. For this reason, the first action after the media provided public education on COVID-19 disease was to establish a Corona National Antivirus Headquarters chaired by the President of Iran and headed by the MOHME. With the establishment of the headquarters, many actions were taken, such as canceling public events and Friday prayers; closing schools, universities, shopping centers and bazaars, as well as holy shrines; and banning festival celebrations. Economic measures were also taken to assist families and businesses. With the intersectoral collaboration, the headquarters is trying to control the outbreak of SARS-CoV-2. The Ministry of Roads and Urban Development initiated the necessary steps for public transport, and the Ministry of Industry, Mine, and Trade will build the required medical equipment. On March 13, 2020, the Revolutionary Guard announced a plan to clear streets, shops, and public places in Iran. In addition, 1,000 fixed and mobile diagnostic clinics will be set up, and the military will work alongside medical providers as well as in the production of face masks and gloves, and army beds will be made available to patients. The Administrative and Employment Affairs Organization has allowed telecommuting of government employee and academic organizations. Academic organizations have also launched learning management systems.

**Fig. 1.**
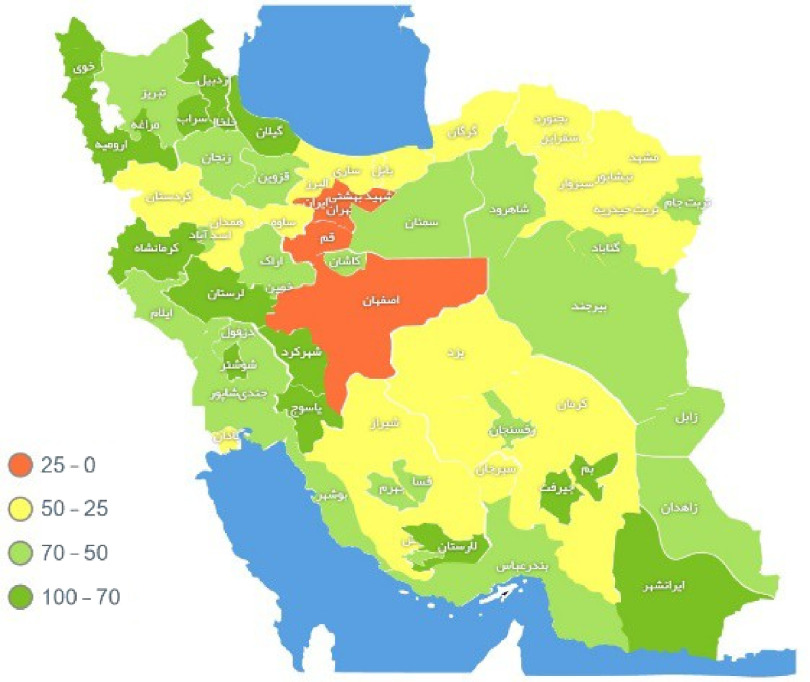
Proportion of the target population screened for COVID-19 by province. **Reference**: *Daily Situation Report on COVID-19, Ministry of Health and Medical Education, IR Iran*.

The government created a mobile software application and a website to battle the COVID-19 epidemic.^9^ The COVID-19 Self-Assessment and Electronic Screening System was launched by MOHME on March 4, 2020 (salamat.gov.ir). When residents log into this system, they provide information such as national code, date of birth, and phone number, and they answer some questions about COVID-19 symptoms and their physical condition. After answering these questions, if a person is suspected of having a coronavirus, follow-up is provided through healthcare centers affiliated with the MOHME. The chart map shows proportion of the target population screened for COVID-19 by province (Fig. 1). People suspected of having COVID-19 receive a text message about their health status. If they do not recover physically after 3 days, they are referred to a hospital. Also, their homes will be disinfected, and other family members will be isolated if needed. The plan has been implemented, and thus far, with the allocation of >17,000 health houses and >9,000 comprehensive health centers in urban, suburban, and rural areas throughout the country. Organized epidemic response work has been carried out at these mobilization centers and bases.

The Social-Law Enforcement Committee of the Coronavirus Battle National Headquarters at the Iranian Interior Ministry began implementing the social distancing plan in March; it will continue through April 2020, and it will be extended by the Committee if necessary. According to the plan, intercity trips will be banned and only locals will be allowed to enter cities and towns. Also, any sites that might draw large clusters of people, including schools, universities, shopping centers, parks, swimming pools, tourist sites, promenades, etc, will be closed. Holding any official or unofficial celebration that can draw crowds will also be forbidden during this period. Maximum restrictions will also be carried out regarding transport by aircraft, trains, and buses. There has been a significant effort to treat COVID-19 patients.^10^ On March 26, 2020, Iran launched plasma therapy for corona-infected patients.^11^ The blood plasma of people who have recovered from COVID-19 infection will be donated to patients to boost their immune systems. Based on the concept of passive immunity, this new method will help improve the condition of patients. In the fight against SARS-CoV-2, it is crucial that countries around the world take steps to prevent transmission and save human lives. The Iranian authorities are implementing their policies and plans with the help of intersectoral collaboration and public participation. However, they should continue to develop new policies and programs to prevent and control the spread of SARS-CoV-2 until a vaccine or medication is available.
